# Effects of Spring Drought and Nitrogen Addition on Productivity and Community Composition of Degraded Grasslands

**DOI:** 10.3390/plants12152836

**Published:** 2023-07-31

**Authors:** Shaoning Li, Shaowei Lu, Xiaohui Li, Xingchen Hou, Xi Zhao, Xiaotian Xu, Na Zhao

**Affiliations:** 1 Institute of Forestry and Pomology, Beijing Academy of Agriculture and Forestry Sciences, Beijing 100093, China; lishaoning@126.com (S.L.); hblsw8@163.com (S.L.);; 2 Beijing Yanshan Forest Ecosystem Positioning Observation and Research Station, Beijing 100093, China; 3 College of Landscape Architecture, Beijing University of Agriculture, Beijing 102203, China; 4 Huamugou Forest Farm, Hexigten Banner, Chifeng City, Inner Mongolia Autonomous Region, Chifeng 025350, China

**Keywords:** degraded grassland, extreme drought, nitrogen addition, aboveground biomass, vegetation composition

## Abstract

To explore whether there were differences among the patterns of response of grasslands with different levels of degradation to extreme drought events and nitrogen addition, three grasslands along a degradation gradient (extremely, moderately, and lightly degraded) were selected in the Bashang area of northern China using the human disturbance index (HDI). A field experiment with simulated extreme spring drought, nitrogen addition, and their interaction was conducted during the growing seasons of 2020 and 2021. The soil moisture, aboveground biomass, and composition of the plant community were measured. The primary results were as follows. (1) Drought treatment caused soil drought stress, with moderately degraded grassland being the most affected, which resulted in an 80% decrease in soil moisture and a 78% decrease in aboveground biomass. The addition of nitrogen did not mitigate the impact of drought. Moreover, the aboveground net primary production (ANPP) in 2021 was less sensitive to spring drought than in 2020. (2) The community composition changed after 2 years of drought treatment, particularly for the moderately degraded grasslands with annual forbs, such as *Salsola collina*, increasing significantly in biomass proportion, which led to a trend of exacerbated degradation (higher HDI). This degradation trend decreased under the addition of nitrogen. (3) The variation in drought sensitivities of the ANPP was primarily determined by the proportion of plants based on the classification of degradation indicators in the community, with higher proportions of intermediate degradation indicator species exhibiting more sensitivity to spring drought. These findings can help to provide scientific evidence for the governance and restoration of regional degraded grassland under frequent extreme weather conditions.

## 1. Introduction

The grassland ecosystem accounts for approximately 40% of China’s land area and has important ecological functions and economic values [1]. Global change, climate drought, nitrogen deposition, land use, and other factors will profoundly change the structure and function of grassland ecosystems [2]. Grassland is usually limited by both water and nutrients [3], and its biomass and community composition are sensitive to changes in water and nitrogen availability [4,5]. Drought directly inhibits plant growth by reducing soil moisture [6,7] and can also indirectly inhibit plant growth and reproduction by reducing the rates of mineralization and contents of the soil’s available nutrients [8,9], which causes changes in its ecosystem structure [10,11]. Moreover, the enrichment in nitrogen caused by its deposition or addition has widely affected the structure and function of terrestrial ecosystems [12], including the promotion of plant growth and accumulation of nitrogen, a reduction in plant community diversity, increase in species dominance, changes in the community composition, and other aspects [13,14,15,16]. Therefore, in the context of the simultaneous occurrence of extreme drought and nitrogen enrichment, studies on the interactive effects of drought and nitrogen addition will help to better understand the responses of soil–plant systems in grasslands to profound changes in global environmental factors.

The effects of drought and the addition of nitrogen to grassland vegetation involve complex coupling effects of water and nutrients. Thus, the combination of the two may not produce a simple additive effect, which can cause inconsistencies in existing research conclusions. The first type of research suggested that the addition of nitrogen made the grassland more susceptible to extreme drought events. Studies on both North American [17] and Chinese temperate grasslands [18] showed that nitrogen fertilization reduced the resistance and temporal stability of grassland aboveground net primary productivity (ANPP) to drought but improved its resilience after drought. A second type of study suggested that the addition of nitrogen did not alter the effect of extreme drought, which was observed in North America [19], the Euro-Mediterranean [20], and temperate grasslands in China [21]. The third type of study found that the effects of nitrogen addition on the drought sensitivity of the grassland ecosystem varied with the severity of drought and the grassland plant community. For example, Shi et al. [22] found that the addition of nitrogen could alleviate the negative effects of short-term drought in a northeastern China meadow grassland, but long-term drought intensified the inhibition on grassland plant communities.

Since large areas of grassland in China are degraded to varying degrees [23], the exacerbation of drought could change the structural attributes of degraded grasslands, which leads to more serious degradation [24]. Moreover, the addition of nitrogen is a common measure used in restoring grassland vegetation, although it is accompanied by some uncertainty of the composition of vegetation. On the one hand, it helps to improve the productivity of vegetation [14] but, alternatively, it leads to a reduction in the diversity of species [12]. Under different climatic conditions, the trends of responses of different plant species and functional types to drought [25] and nitrogen enrichment [26] were not consistent. For example, grasses are generally more resistant to drought than forbs [25,27] and benefit more from enrichment by concentrated nitrogen [28]. Changes in community composition caused by grassland degradation may regulate the effects of drought and nitrogen addition [16,29,30]. Therefore, elucidating the abilities of dominant grassland species in grasslands with different levels of degradation to respond to drought, and their mechanism of response to nitrogen addition, is very important for grassland restoration in the future context of complex global change.

Based on these scientific facts, this study selected grassland fields located in the same region, but with community compositions owing to different levels of degradation in northern China, and primarily explored their different performances in response to simulated drought during the early stages of growth (designated “spring drought” from this point onward) under the condition of added nitrogen and the effect of additional fertilization. The resistance to climate drought during the process of restoring degraded grasslands was explored. This study aimed to answer the following questions. (1) How does the interaction between spring drought and nitrogen addition affect biomass and the composition of vegetation of degraded grassland? (2) How does the composition of vegetation regulate its responses to spring drought and nitrogen addition at different levels of grassland degradation?

## 2. Results

### 2.1. Responses of Soil Moisture

**Figure 1:**
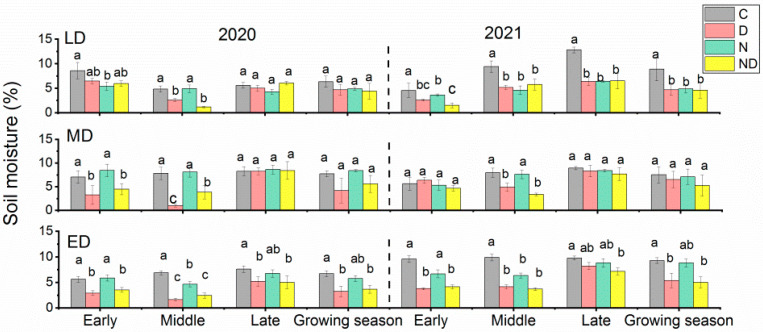
Soil moisture of grasslands with different degradation statuses during the growing seasons of 2020 and 2021. Early, middle, and late represent the monthly mean values of soil moisture from May 15 to June 15, June 15 to July 15, and July 15 to August 15, respectively. Growing season represents the mean values of soil moisture from May 15 to August 15. Bars with the same lowercase letters were not significantly different in Duncan tests (α = 0.05). C, control; D, drought; ED, extremely degraded grassland; LD, lightly degraded grassland; MD, moderately degraded grassland; N, nitrogen addition; ND, nitrogen addition under drought treatment.

Compared with the natural control, spring drought treatments caused a significant decrease in soil moisture in all three fields (*p* < 0.05). During drought treatments, the soil moisture of the lightly degraded grassland decreased by 11~23%; that of the moderately degraded grassland decreased by 13~80%; and that of the extremely degraded grassland decreased by 44~58% (*p* < 0.05). (Early and Middle stages in Figure 1). The soil moisture of moderately degraded grasslands recovered to the same level as that of the control group at one month after the drought treatments had been completed (Late in Figure 1), while the soil moisture of lightly degraded grassland in 2020 and extremely degraded grassland in 2021 was still significantly lower than that of the control (*p* < 0.05) (Figure 1). As for the mean soil moisture during the growing season, there were generally insignificant drought effects for lightly and moderately degraded grasslands, primarily owing to the smoothing effects of the soil moisture during the rewatering stages (Late in Figure 1). The interactions between spring drought and nitrogen addition resulted in a further decrease in soil moisture in different degraded grasslands. During the drought treatments, the soil moisture in the interactive treatment group was significantly lower than those in the nitrogen addition group (*p* < 0.05) in the lightly, moderately, and extremely degraded grasslands in 2020, and in the moderately and extremely degraded grasslands in 2021. Compared with the natural control, there was a similar decrease in soil moisture in the interactive treatment group and the spring drought group (*p* > 0.05). Therefore, treatment with added nitrogen did not change the effect of drought on soil moisture (Figure 1).

### 2.2. Responses of ANPP

**Figure 2:**
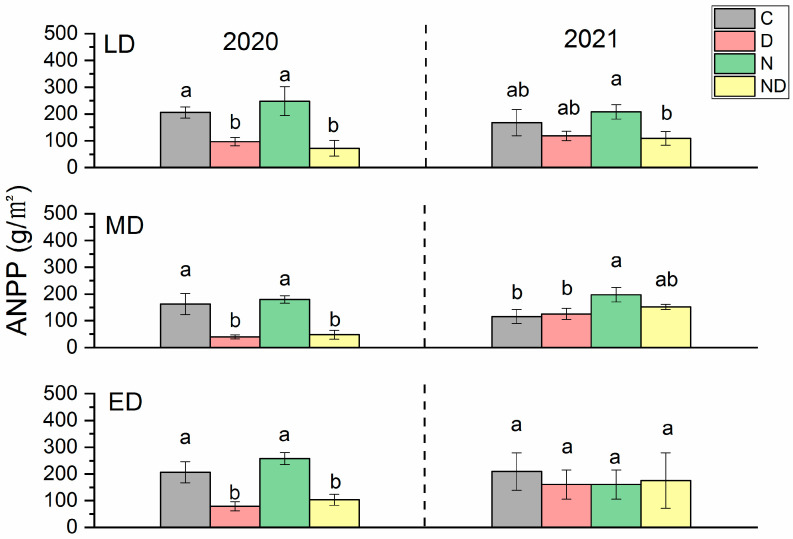
ANPP of grasslands with different degradation statuses during the growing seasons of 2020 and 2021. Bars with the same lowercase letters were not significantly different in Duncan tests (α = 0.05). C, control; D, drought; ED, extremely degraded grassland; LD, lightly degraded grassland; MD, moderately degraded grassland; N, nitrogen addition; ND, nitrogen addition under drought treatment.

Compared with the natural control, the ANPP under extreme drought treatment in 2020 decreased significantly (*p* < 0.05) across all the fields, and the lightly, moderately, and extremely degraded grasslands decreased by 36%, 78%, and 62%, respectively. In 2021, extreme drought treatment did not cause a significant decrease in ANPP (*p* > 0.05) (Figure 2). The addition of nitrogen increased the ANPP, but there was no significant difference between the treatment of added nitrogen and the natural control group (*p* > 0.05) except for the moderately degraded grassland in 2021 (*p* < 0.05). The effects of interactive treatment of extreme drought and the addition of nitrogen varied between both years. In 2020, the ANPP of lightly, moderately, and extremely degraded grasslands were significantly reduced by 63%, 76%, and 51% (*p* < 0.05), respectively, which was similar to that of the extreme drought treatment group. In 2021, the interactive treatment of spring drought and nitrogen addition only significantly reduced the ANPP of lightly degraded grasslands (*p* < 0.05) but did not significantly change the ANPP of moderately and extremely degraded grasslands (*p* > 0.05) (Figure 2).

**Figure 3:**
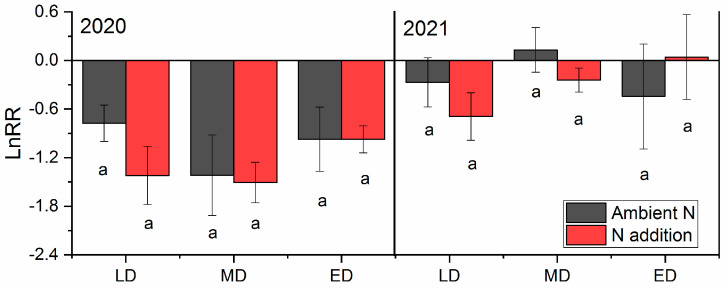
Natural logarithm response ratio of the ANPP of grasslands with different degradation statuses during the growing seasons of 2020 and 2021. Bars with the same lowercase letters were not significantly different in Duncan tests (α = 0.05). ANPP, aboveground net primary production; C, control; D, drought; ED, extremely degraded grassland; LD, lightly degraded grassland; MD, moderately degraded grassland; N, nitrogen addition; ND, nitrogen addition under drought treatment.

The drought sensitivities of ANPP were not significantly different across all the fields and were also not changed by the addition of nitrogen. Among them, the lightly and moderately degraded grasslands showed a tendency to be more sensitive to drought following the treatments of added nitrogen with lower lnRR values of the conditions of added nitrogen than those of ambient nitrogen. However, these differences were not statistically significant (*p* > 0.05). In contrast, the drought sensitivity of ANPP showed inter-annual differences, and the lnRR values in 2020 (−1.51~−0.77) were significantly lower than those in 2021 (−0.44~0.13) (*p* < 0.05), indicating that the ANPP was less sensitive to drought during the second year of treatment (Figure 3).

### 2.3. Responses of the Composition of Vegetation from the Functional Groups and Degradation of the Indicator Species

**Figure 4:**
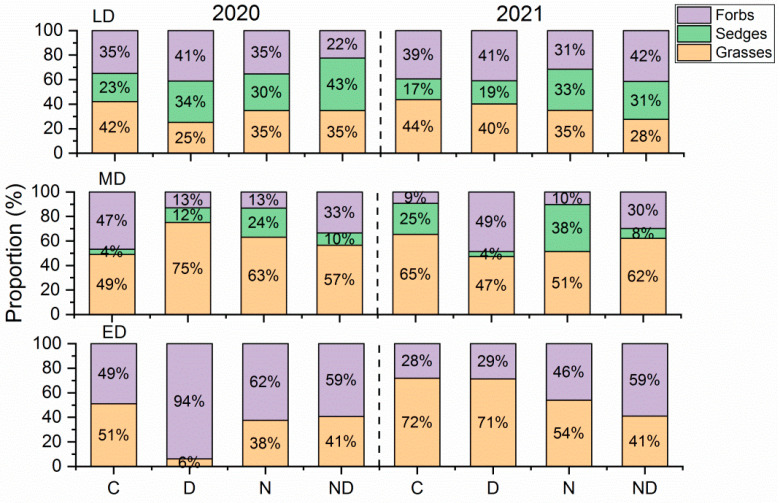
Proportion of the ANPP of functional groups based on grasses, sedges, and forbs. ANPP, aboveground net primary production; C, control; D, drought; ED, extremely degraded grassland; LD, lightly degraded grassland; MD, moderately degraded grassland; N, nitrogen addition; ND, nitrogen addition under drought treatment.

In terms of the proportion of plant functional groups based on grasses, sedges, and forbs, extreme drought and added nitrogen had no significant effect on grasslands with different levels of degradation (Figure 4). The proportion of functional groups in the lightly degraded grassland was relatively stable and ranged from 17% to 44%, while the proportion of grasses decreased from 42~44% to 25~40%. The proportion of grasses was not significantly affected by extreme drought and treatment with added nitrogen (*p* > 0.05). Extreme drought reduced the proportion of sedges in the moderately degraded grassland (*p* < 0.05), while there were almost no sedges in the extremely degraded grassland (Figure 4, Table S1).

**Figure 5:**
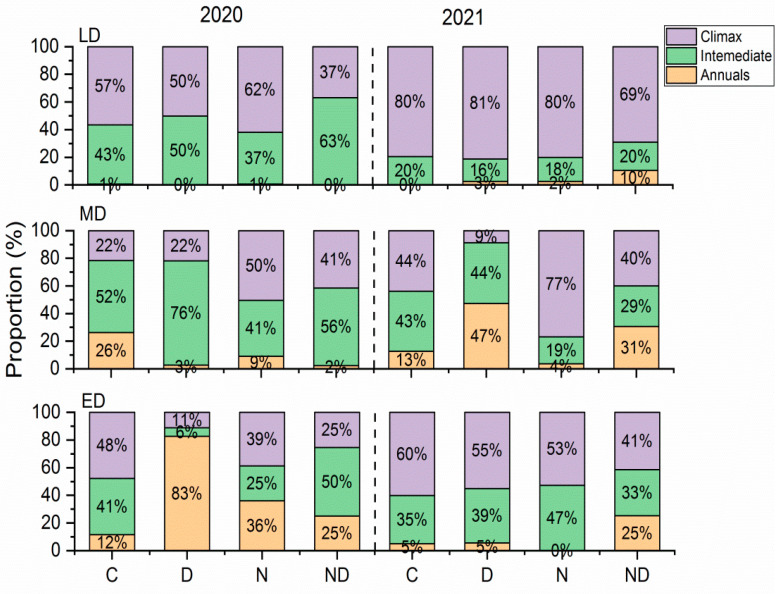
Proportions of ANPP of the degradation indicators. ANPP, aboveground net primary production; C, control; D, drought; ED, extremely degraded grassland; LD, lightly degraded grassland; MD, moderately degraded grassland; N, nitrogen addition; ND, nitrogen addition under drought treatment.

In terms of the proportion of degraded indicator plants, the effects of extreme drought and treatment with nitrogen on the grasslands with different levels of degradation varied (Figure 5). The proportion of degradation indicator species in the lightly degraded grassland was relatively stable, and the proportion of climax species remained > 50% (except for the ND treatment in 2020), and there was no significant difference among the different treatments (*p* > 0.05). Extreme drought reduced the proportion of climax species in the moderately degraded grassland from 22–44% to 9–22%, while the addition of nitrogen increased the proportion of climax species to 50–77% (*p* < 0.05). Extreme drought treatment also decreased the proportion of climax species from 48–60% to 11–55% in extremely degraded grassland, but the addition of nitrogen did not significantly increase the proportion of climax species (*p* > 0.05). Compared with 2020, the proportion of intermediate degradation indicator species in both the lightly and moderately degraded grasslands decreased in 2021, while the proportion of intermediate degradation indicator species in the extremely degraded grasslands showed no significant trend (Figure 5, Table S1).

### 2.4. Relationships between ANPP Drought Sensitivity, the Composition of Vegetation of Plant Functional Groups, and Degradation Indicator Species

**Figure 6:**
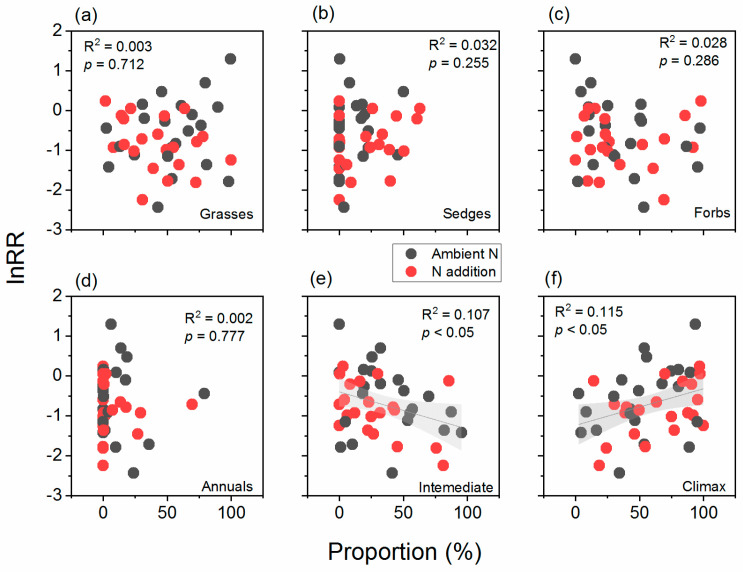
Relationships between drought sensitivity (natural logarithm response ratio, lnRR) of aboveground net primary production and plant functional group compositions of (**a**) grasses, (**b**) sedges, and (**c**) forbs, and the degradation indicator species of (**d**) annuals, (**e**) intermediate degradation indicators, and (**f**) climax species.

A regression analysis showed that there were no significant relationships between the proportions of functional groups of grasses, sedges, forbs, and annuals and the drought sensitivity of ANPP (Figure 6a–d). However, the lnRR values of ANPP decreased significantly with the increase in proportion of intermediate degradative indicator species (R^2^ = 0.107) (Figure 6e) and significantly increased with the increase in proportions of climax species (R^2^ = 0.115) (Figure 6f) (*p* < 0.05). Therefore, grasslands with a high proportion of intermediate degradation indicator species will be more sensitive to drought, while grasslands with a high proportion of climax species will be less sensitive. However, the addition of nitrogen had no significant effect on the trends described above (*p* > 0.05). The multiple regression analysis that contained all the plant functional groups showed an improved R^2^ of 0.128, but the model was not statistically significant (*p* > 0.05) (Table S2).

## 3. Discussion

### 3.1. Effects of the Interactions of Spring Drought and Nitrogen Addition on the ANPP of Degraded Grasslands

In grassland ecosystems, extreme drought often coincides with other drivers of chronic environmental change, and nitrogen enrichment is one of the most significant drivers [12,31]. Enrichment with nitrogen can relieve the limitation of soil nutrients and improve plants’ effectiveness at utilizing precipitation [32], thus, improving the ANPP [16]. However, in semiarid ecosystems represented by grassland, the availability of water is a primary factor that limits the productivity of plant community [33]. When extreme drought events of up to 60 days occur, the dramatic decrease in soil moisture will cause drought stress in the soil, which renders water the minimum limiting factor. Therefore, additional supplementation with nutrients cannot mitigate the negative effects of drought on the ANPP of degraded grasslands. Consistent with the results of most studies [25,31], this study found that extreme drought treatment significantly reduced the amount of soil water in different degraded grasslands during the drought period (*p* < 0.05), while the addition of nitrogen did not alleviate the soil water stress caused by extreme drought events. Friedrich et al. [34] in Germany and Shi et al. [22] in northeast China both found that the addition of nitrogen did not significantly affect the decrease in soil moisture caused by extreme drought, nor did it change the drought sensitivity of grassland vegetation. Some studies suggested that the addition of nitrogen can lead to a disproportionate increase in the aboveground biomass by promoting plant growth and increasing evaporation, which leads to more water loss, thus exacerbating the amount of drought stress in the soil [17].

Unlike previous studies, this one identified significant differences in the drought sensitivity of degraded grasslands between the two years. After experiencing extreme drought treatment in the first year (2020), the ANPP was significantly less sensitive to re-drought in the following year (2021). This indicated that the degraded grassland vegetation in this study had “drought memory” [35], which indicated that a previous drought can increase the resistance of vegetative biomass to drought, which manifests as a positive “legacy effect” [6]. As the current hotspot and difficulty in global change ecology, drought legacy effects have complex characteristics [36]. Grasslands generally show positive legacy effects and become more resistant to drought as the number of droughts increases. There are two primary reasons for this. First, the drought resistance of plants can be improved after drought. For example, Li et al. [37] found that under repeated drought conditions, the content of proline in Chinese rye grass (*Leymus chinensis*) increased, and thus its adaptability to drought was improved by regulating osmotic pressure. Secondly, drought affected the composition of plant communities and increased the proportion of plant functional groups that are highly resistant to drought. For example, grasses are usually highly resistant to drought, and drought will promote an increase in the proportion of grasses [25,29]. However, continuous multiple extreme droughts may break the critical value of plant tolerance to drought stress, which leads to negative legacy effects [25,38].

### 3.2. Regulation of Drought Sensitivity of Degraded Grassland by Plant Functional Groups and Degradation Indicator Species

Previous studies have confirmed that extreme drought treatment can change the composition of plant functional groups to some extent [39], and the plant community composition can affect the impact of drought [29]. In this study, compared with the first year, the proportion of climax species and grasses in the lightly and extremely degraded fields generally increased during the second year of drought, while the proportion of annuals and forbs in the moderately degraded fields generally increased. A previous study showed that climax grasses, such as *L. chinensis*, and annual forbs, such as *Salsala collina*, are both species that are highly resistant to drought [27]. In contrast, the proportion of the composition of functional groups in lightly degraded grassland in this study was relatively stable during both years, which indicated that the vegetation community of grassland that had a highly diverse group of plants and better soil conditions was more effective at buffering environmental change. The increase of annuals and forbs in moderately degraded grassland aggravated the degradation of species composition. This is consistent with previous studies; the occurrence of drought will enhance the degradation of grassland [24].

The changes in the functional groups of grassland plants after drought were primarily owing to the different responses of various dominant plant species to drought stress, and this change further affected the drought sensitivity of ANPP. Among the plant functional groups, grasses are generally considered to be strongly resistant to drought, and their dominance increased under drought conditions, while forbs showed the opposite trend [25,29,40]. However, this phenomenon was not always true [41]. In this study, the relative proportions of grasses and forbs were shown to be insensitive to both extreme drought and the addition of nitrogen (Figure 4), while the plant classifications based on degradation indicator species showed a closer relationship with the drought sensitivity of the grassland ANPP (Figure 5). Facing extreme drought events as disturbance, herb species can adopt three strategies based on Grime’s C-S-R (Competitor-Stress Tolerator-Ruderal) framework [42]. Among grasses, the rhizomatous species, which are represented by *L. chinensis*, can sprout via underground rhizomes after the aboveground part dies owing to environmental stress [43], showing a competitor strategy. Alternatively, annuals, such as green foxtail (*Setaria viridis*) and *Sa. collina*, can quickly complete their life cycle through seed germination after drought and rewatering [44], showing a ruderal strategy. Therefore, there were relatively low responses from the grasses and annuals to interannual drought sensitivities. In contrast, some typical perennial intermediate degradation indicator species, such as *Cleistogenes squarrosa* and *Artemisia frigida*, tended to adopt tolerant strategies under drought conditions [25,42], showing a stress tolerator strategy. However, stress tolerators had difficulty adapting to extreme drought events that exceeded their limits of tolerance [45]. This framework can partly explain the regulation of the proportions of degradation indicators and climax species on the drought sensitivities of ANPP. Given that the R^2^ to explain this relationship was relatively low, we call for experiments with a longer duration and more detailed observations on drought sensitivity at species level on this issue. Simultaneously, the addition of nitrogen changed the relative dominance of plants with different strategies of acquiring nitrogen [46]. Previous studies have shown that the addition of nitrogen exacerbated the negative effects of long-term drought [22]. This study further confirmed that under the conditions of nitrogen addition, the effect of this phenomenon was more apparent in grasslands that contained a high proportion of intermediate degraded indicator species (Figure 6e).

### 3.3. Suggestions on Grassland Restoration and Management

Based on the results described above, this study proposed the following suggestions to restore and manage degraded grassland. (1) The grassland plants in the study area primarily grew rapidly from May to July [47]. The extreme drought events in the early and middle periods of this study covered this period, which would have a serious impact on the grassland ANPP. Therefore, more stringent grazing bans should be implemented on degraded grasslands during this period to prevent the compounding effects of drought from causing more degradation. (2) With the change in level of grassland degradation, the responses of various plant functional groups to extreme drought can be different, and dividing the community based on degradation indicator species can better indicate the effects of extreme drought than those based on grasses, sedges, and forbs [27]. Therefore, future grassland restoration and management to improve the quality of grassland should increase the monitoring of and research on degradation indicator species. (3) The relative proportion of intermediate degradative indicator species and climax species was found to determine the drought sensitivity of ANPP. Therefore, future grassland restoration should proceed by increasing the proportion of climax species by targeted reseeding plants that can more effectively improve the ability of degraded grassland to respond to extreme drought events. (4) Currently, supplementing the soil nutrients of grassland by applying nitrogen fertilizer is an effective measure to restore degraded grasslands [3,16]. However, its effect can be altered by extreme drought events, and in grasslands with a high proportion of intermediate degradation indicator species, the application of nitrogen would result in a more serious drought effect. All types of improvement measures are somewhat ecologically adaptable. Thus, appropriate measures should be selected based on the factors such as climatic conditions, human utilization, level of degradation, and the restoration target of grasslands.

## 4. Conclusions

This study explored the responses of grasslands with different levels of degradation to extreme drought and the addition of nitrogen to the content of their soil water, ANPP, and composition of plants. The results showed that under extreme drought conditions, treatment with additional nitrogen could not alleviate the decrease in ANPP caused by soil water stress. However, the composition of vegetation functional groups in degraded grasslands that have experienced an extreme drought event changed, which further reduced their sensitivity to subsequent drought. This effect was primarily determined by the proportion of degraded indicator plants in the community. The community with higher proportions of intermediate degradation indicator species was more sensitive to extreme drought. For moderately degraded grassland, after two consecutive years of drought treatment, the community composition changed and annual forbs, such as *Sa. collina*, increased significantly. This study revealed the regulation of composition based on degradation indicator species by the response of degraded grassland to extreme drought and added nitrogen, indicating that it is important to systematically understand the response of different functional groups of plants in various dominant species ecosystems to future extreme climate in more detail from the perspective of degradation of the composition of grassland communities.

## 5. Materials and Methods

### 5.1. Study Areas

The study site is located in Weichang Manchu and Mongol Autonomous County, Chengde City, Hebei Province, China, with an altitude of approximately 1500 m (Figure S1). It is a transitional zone from humid to arid climates with climates that fluctuate a great deal and undergo intense environmental changes, and it is also a marginal area of ecologically fragile forest distribution, with an annual precipitation of approximately 400 mm. The area is an intermingled zone of agriculture, forestry, and pastoralism, with a high intensity of anthropogenic activities, which leads to drastic ecological degradation owing to unreasonable reclamation and grazing [48].

### 5.2. Sample Site Selection

At the beginning of 2019, three flat grasslands with a slope of <5° were selected as the experimental sites in the Yudaokou pasture (42°19′3″ N, 117°12′3″ E), and the distance between the sites was ≤2 km to ensure that the environmental conditions, such as climate and original vegetation types, were basically the same. Each sample field was at different levels of degradation owing to the different intensities of human utilization. Therefore, based on the research of Liu et al. [49], the human disturbance index (HDI) values were calculated to quantify the level of grassland. The three fields were divided into extremely, moderately, and lightly degraded grassland. The formula for calculation was as follows:

(1) $$HDI=1/\left(A_{1}\times \frac{1}{3}+A_{2}\times \frac{2}{3}+A_{3}\right)$$

where $A_{1}$, $A_{2}$, and $A_{3}$ represent important values of the annuals, such as *Sa. collina* and *Chenopodium aristatum*; perennial intermediate degradation indicator species, such as *Potentilla bifurca* var. *major* and *Stellera chamaejasme*; and perennial climax species, such as *L. chinensis* and *Potentilla longifolia*, respectively. Their weights accounted for one-third, two-thirds, and one, respectively. Higher HDI values indicate more severe levels of degradation. As previously concluded, these three groups of herb species primarily appeared in the serious, moderately, and lightly or non-degraded grasslands in northern China [49,50,51]. Based on a previous comprehensive study of vegetation in this region, this classification primarily reflects the reverse succession sequence of vegetation community as the degradation of grassland became exacerbated [50,51]. The major degradation indicator species are listed in a study by Zhao et al. [52], and the inventories of species in the three fields during 2020 and 2021 are documented in Table S3.

(2) $$A_{i}=\left({RH}_{i}+{RA}_{i}+{RC}_{i}\right)/3$$

where *RH_i_*, *RA_i_*, and *RC_i_* represent the relative height, relative coverage, and relative abundance of species i, respectively.

The basic information of the sample fields was as follows.

*ED, extremely degraded grassland*. The site was heavily deserted owing to long-term overgrazing with very low species richness. In terms of vegetation composition, although there were climax species, such as *L. chinensis*, and annual plants, such as sand rice (*Agriophyllum squarrosum*), *Se. viridis*, dominated. In addition, there were also indicators of degradation, such as *Agropyron mongolicum* var. *villosum* and *P. bifurca* var. *major*. There was more than 90% of sand in the soil surface, and the content of soil nitrogen was 0.022 ± 0.004%. The HDI was 1.49 ± 0.10.

*MD, moderately degraded grassland*. The practice of long-term grazing resulted in relatively light degradation of the soil surface. The primary species included climax species, such as *L. chinensis* and *P. longifolia*, and degradation indicator species, such as *C. squarrosa* and *P. bifurca* var. *major*, that were present at high levels, and annuals that were present at low levels. The contents of sand in the soil and its nitrogen were between those of the extremely and lightly degraded grassland. The HDI was 1.21 ± 0.03.

*LD, lightly degraded grassland*. This sample field was a grazing pasture under human management with low grazing pressure and a high richness of species. The primary species were climax species (*L. chinensis*, *P. longifolia*, and *Iris tenuifolia*, among others), and there was a relatively low ratio of degradation indicator species (*C. squarrosa* and *A. frigida*, among others) and annual plants. There was <70% sand in the soil, and the content of nitrogen was 0.143 ± 0.016%. The HDI was 1.09 ± 0.02.

### 5.3. Experimental Design

The same experiments were conducted in three selected fields with different levels of degradation for horizontal comparison. The experimental treatments were randomly distributed in 2 m × 2 m experimental samples with 4 m buffer zones within and between the groups. There were four treatments (control, spring drought treatment, nitrogen addition treatment, and spring drought and nitrogen addition interactive treatment), four replicates, and three fields with a total of 48 plots (Figure S1). A 50 cm depth of soil was dug up by trenching around each plot, and impervious plastic sheets were buried. Soil ridges that were 10 cm high were built around the plots to prevent the lateral flow of water.

In this study, shelters that reduced the access of rain to the plots were used to simulate extreme drought. These types of shelters that reduce rain are widely used in grassland ecological experiments, with the roofs constructed of a 2.3 m × 2.2 m rain shelter (polycarbonate with 90% light transmittance) above iron scaffoldings with windward and leeward heights of 1.5 m and 1.2 m, respectively, and the area of the rain shelters was slightly larger than that of the plots. Transparent plastic cloth sheets were set up under the roofs to prevent the transverse wind from blowing rain into the plots, and a height of more than 50 cm near the ground was set aside for ventilation without hindering the growth of plants or the entrance of observers. Previous studies have found that local grasslands usually start to turn green in May, grow rapidly in June, reach their peak growth in mid-to-late July, and then begin to decline [47]. Thus, this experiment was designed to focus the effects of the drought from mid-May to mid-July. Mid-May to mid-June was defined as the early growing season, mid-June to mid-July was the middle growing season, and the late growing season was mid-July to mid-August when the shelters that reduced rain were removed.

The experimental design of nitrogen addition included two treatments: the control group (N0, 0 g N m^−2^ yr^−1^) and the nitrogen addition group (N10, 10 g N m^−2^ yr^−1^), which both interacted with the spring drought treatments (drought and no-drought). The rate of application of the nitrogen addition treatment was based on the nitrogen saturation values (10–20 g N m^−2^ yr^−1^) and the maximum response values (<20 g N m^−2^ yr^−1^) of the contents of phytoliths found in previous studies [16,53,54]. Considering the safety and availability and the exclusion of interference from other elements, such as phosphorus, urea was selected as the type of fertilizer. The plots were fertilized in early May, June, July, and August each year. The method of fertilization was solution spraying, in which the control group was also given the same amount of water as the nitrogen group. Approximately 0.3 L per month of water was added, which was equivalent to 0.08 mm of monthly precipitation. This was much less than the local natural precipitation. Thus, it could be considered that it would not have a significant impact on the water supply of the grassland.

### 5.4. Data Collection

*Soil moisture measurement*: during the study, a plastic measuring tube with a closed bottom was placed right in the center of each experimental plot. The soil moisture of 0–20 cm in the plot was measured weekly using a TDR soil moisture meter (IMKO, Ettlingen, Germany).

*Biomass measurement*: when the biomass reached its peak in mid-August of each year, the samples were harvested at ground level using a 0.5 m × 0.5 m sampling frame and divided into green plants and standing litter. For the plot of 2 m × 2 m, 30 cm was left on the edges for buffer zones where no sampling or studies were conducted, and the sampling positions were selected randomly yearly around the plot center, which was the position of the TDR tube. The green plants were further divided into functional groups, including grasses, sedges, and forbs, and degraded indicator species, including annuals, intermediate degradation indicator species, and perennial climax species, and the sampling subplots were rotated once a year. The information about the litter biomass is shown in Table S4.

*Vegetation composition survey*: detailed species surveys were conducted at fixed positions within the plots (excluding the buffer zones) with a frame of 0.5 m × 0.5 m. A survey of the height (in average in the community), coverage (fractional projected area), and abundance (number of individuals) of all species was conducted in early August before the annual biomass was harvested to analyze the grassland degradation index.

### 5.5. Statistical Analysis

This study utilized analysis of variance (ANOVA) based on the result of general linear model (GLM), post hoc Duncan’s tests to analyse the statistical differences in soil moisture, and aboveground biomass among all the treatments (control, spring drought, nitrogen addition, and the interaction treatment), with a statistical significance level of *p* < 0.05, where treatment was a fixed factor and block was a random factor. A Levene test was used to test the homogeneity of variance of the samples.

Natural log response ratios (lnRRs) of the ANPP were calculated to represent its drought sensitivities [55]:

lnRR = ln(ANPP_T_) − ln(ANPP_C_) (3)

where ANPP_T_ and ANPP_C_ are the ANPP of treatments and the ANPP of control groups, respectively.

Linear regressions were conducted to analyze the relationships between the proportions of vegetation functional groups and drought sensitivity of ANPP. SPSS 21.0 (IBM Corp., Armonk, NY, USA) was used for all the data analyses in this study, and Origin 2018 (OriginLab, Northampton, MA, USA) was used to draw all the graphs.

## Data Availability

The data that support the findings of this study are available on request from the corresponding author.

## Supplementary Materials

The following supporting information can be downloaded at: https://www.mdpi.com/article/10.3390/plants12152836/s1, Figure S1. Distribution of grassland in norther China and the geographical location of the study site (a), and the layout of experimental design (b). C = control, D = drought, N = Nitrogen addition, and ND = Nitrogen addition under drought treatment; Table S1. Standard errors of proportions of ANPP of functional groups and degradation indicators of grasslands with different degradation status during growing seasons of 2020 and 2021; LD = lightly degraded grassland, MD = moderately degraded grassland, and ED = extremely degraded grassland; C = control, D = drought, N = Nitrogen addition, and ND = Nitrogen addition under drought treatment; Table S2. Results of the multiple regression analysis containing all of the plant functional groups and degradation indicator species; Table S3. Inventory of degradation indicator species in the study fields; Table S4. Standing litter biomass of grasslands with different degradation status during growing seasons of 2020 and 2021; LD = lightly degraded grassland, MD = moderately degraded grassland, and ED = extremely degraded grassland; C = control, D = drought, N = Nitrogen addition, and ND = Nitrogen addition under drought treatment.
