# Supplementary material for: Effects of Spring Drought and Nitrogen Addition on Productivity and Community Composition of Degraded Grasslands
Source: Plants (Basel). 2023 Jul 31;12(15):2836. doi: 10.3390/plants12152836 (PMC10421370; doi:10.3390/plants12152836)
Supplement: Supplementary file 1 [file plants-12-02836-s001.zip › plants-2474143-supplementary.pdf]

Effects of spring drought and nitrogen addition on productivity and community composition of degraded grasslands

LI Shao-ning[1, 2, 3, #], LU Shao-wei[1, 2, 3, #], LI Xiao-hui[4], HOU Xing-chen[1, 2, 3], ZHAO Xi[1, 2, 3], XU Xiao-tian[1, 2, *], ZHAO Na[1, 2, *]

[1] Institute of Forestry and Pomology, Beijing Academy of Agriculture and Forestry Sciences, Beijing 100093, China;
[2] Beijing Yanshan Forest Ecosystem Positioning Observation and Research Station, Beijing 100093, China
[3] College of Landscape Architecture, Beijing University of Agriculture, Beijing 102203, China
[4] Huamugou Forest Farm, Hexigten Banner, Chifeng City, Inner Mongolia Autonomous Region, Chifeng, Inner Mongolia 025350, China
# These authors contributed equally to this work.
* Correspondence: arthurpku@163.com； zhaona1019@126.com; Tel.: 010-62599989

Tables

Table S1. Standard errors of proportions of ANPP of functional groups and degradation indicators of grasslands with different degradation status during growing seasons of 2020 and 2021; LD = lightly degraded grassland, MD = moderately degraded grassland, and ED = extremely degraded grassland; C = control, D = drought, N = Nitrogen addition, and ND = Nitrogen addition under drought treatment.

Field	Treatment	Grasses		Sedges		Forbs		Annuals		Intermediate		Climax	
		2020	2021	2020	2021	2020	2021	2020	2021	2020	2021	2020	2021
LD	C	0.09	0.07	0.13	0.01	0.08	0.07	0.01	0.00	0.12	0.06	0.12	0.06
	D	0.09	0.13	0.13	0.03	0.18	0.16	0.00	0.02	0.16	0.05	0.16	0.06
	N	0.08	0.10	0.15	0.12	0.17	0.08	0.01	0.02	0.23	0.10	0.22	0.11
	ND	0.07	0.08	0.08	0.10	0.10	0.03	0.00	0.07	0.17	0.07	0.17	0.01
MD	C	0.17	0.07	0.02	0.09	0.19	0.02	0.19	0.04	0.14	0.10	0.08	0.06
	D	0.07	0.15	0.04	0.03	0.06	0.17	0.02	0.18	0.10	0.16	0.11	0.04
	N	0.06	0.13	0.06	0.09	0.04	0.05	0.09	0.03	0.14	0.05	0.14	0.06
	ND	0.05	0.11	0.03	0.04	0.06	0.13	0.02	0.12	0.05	0.04	0.07	0.10
ED	C	0.18	0.20	0.00	0.00	0.18	0.20	0.08	0.02	0.22	0.21	0.18	0.20
	D	0.06	0.18	0.00	0.00	0.06	0.18	0.17	0.04	0.06	0.12	0.11	0.10
	N	0.14	0.27	0.00	0.00	0.14	0.27	0.12	0.00	0.09	0.27	0.03	0.27
	ND	0.20	0.23	0.00	0.00	0.20	0.23	0.15	0.25	0.26	0.20	0.12	0.21

Table S2. Results of the multiple regression analysis containing all of the plant functional groups and degradation indicator species.

Argument	B	β	t	P	F	R^2
Intermediate degradation indicators	-0.952	-0.336	-1.929	0.061	1.391	0.128
Grasses	-0.016	-0.006	-0.032	0.974		(P = 0.255)
Sedges	0.317	0.077	0.428	0.671		
Annuals	-0.458	-0.101	-0.561	0.578		

Table S3. Inventory of degradation indicator species in the study fields.

Group	LD	MD	ED
Annuals	*Androsace septentrionalis*<br>*Chamaerhodos erecta*<br>*Chenopodium aristatum*<br>*Salsola collina*<br>*Setaria viridis*	*Artemisia scoparlia*<br>*Chenopodium aristatum*<br>*Eleusine indica*<br>*Salsola collina*<br>*Setaria viridis*	*Salsola collina*<br>*Setaria viridis*
Intermediate degradation indicators	*Agropyron mongolicum*<br>*Artemisia frigida*<br>*Bromus inermis Cleistogenes squarrosa*<br>*Festuca rubra Heteropappus altaicus*<br>*Thymus mongolicus*<br>*Potentilla bifurca* var. *major*	*Agropyron mongolicum*<br>*Artemisia frigida*<br>*Bromus inermis*<br>*Cleistogenes squarrosa*<br>*Festuca rubra*<br>*Thymus mongolicus*	*Agropyron mongolicum*<br>*Artemisia frigida*<br>*Bromus inermis*<br>*Polygonum divaricatum*<br>*Potentilla bifurca* var. *major*
Climax species	*Adenophora elata*<br>*Allium senescens*<br>*Artemisia tanacetifolia*<br>*Artemisia eriopoda*<br>*Carex korshinskii*<br>*Dianthus chinensis*<br>*Galium verum*<br>*Koeleria cristata*<br>*Leontopodium leontopodioides*<br>*Leymus chinensis*<br>*Medicago falcata*<br>*Oxvtropis chiliophylla*<br>*Papaver nudicaule*<br>*Poa sphondylodes*<br>*Potentilla longifolia*<br>*Rumex acetosella*<br>*Schizonepeta tenuifolia*	*Allium senescens*<br>*Artemisia eriopoda*<br>*Bupleurum chinense*<br>*Calamagrostis epigeios*<br>*Carex korshinskii*<br>*Iris tenuifolia*<br>*Koeleria cristata*<br>*Leymus chinensis*<br>*Medicago falcata*<br>*Oxytropis chiliophylla*<br>*Poa sphondylodes*<br>*Potentilla longifolia*<br>*Rumex acetosella*<br>*Thalictrum squarrosum*	*Artemisia tanacetifolia*<br>*Calamagrostis epigeios*<br>*Carex korshinskii*<br>*Leymus chinensis*<br>*Medicago falcata*<br>*Rumex acetosella*<br>*Scutellaria scordifolia*<br>*Thalictrum squarrosum*

Table S4. Standing litter biomass of grasslands with different degradation status during growing seasons of 2020 and 2021; LD = lightly degraded grassland, MD = moderately degraded grassland, and ED = extremely degraded grassland; C = control, D = drought, N = Nitrogen addition, and ND = Nitrogen addition under drought treatment.

	Treatment	2020	2021
LD	C	7.90±3.17	4.14±2.29
	D	4.20±2.47	4.32±2.50
	N	4.20±4.20	31.1±10.90
	ND	3.80±2.34	5.21±3.14
MD	C	1.00±1.00	11.10±1.95
	D	3.40±1.25	4.13±2.44
	N	12.70±6.38	15.54±5.35
	ND	4.10±3.13	9.99±7.62
ED	C	3.90±2.73	4.43±3.71
	D	2.30±1.52	2.25±2.25
	N	6.70±3.82	0.00±0.00
	ND	0.70±0.70	4.97±2.88

Notes: Values with the same lowercase letters were not significantly different in tests of Duncan.

Figures

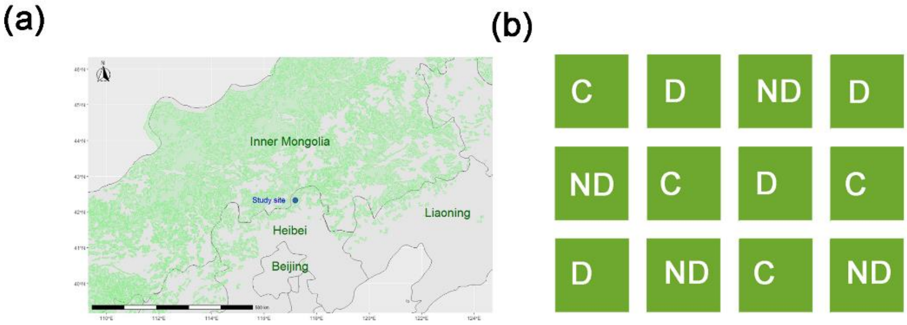

Figure S1. Distribution of grassland in norther China and the geographical location of the study site (a), and the layout of experimental design (b). C = control, D = drought, N = Nitrogen addition, and ND = Nitrogen addition under drought treatment.
